# Clinical Use of Surface Electromyography to Track Acute Upper Extremity Muscle Recovery after Stroke: A Descriptive Case Study of a Single Patient

**DOI:** 10.3390/asi4020032

**Published:** 2021-05-10

**Authors:** Heather A. Feldner, Christina Papazian, Keshia M. Peters, Claire J. Creutzfeldt, Katherine M. Steele

**Affiliations:** 1 Department of Rehabilitation Medicine, University of Washington, Seattle, WA 98195, USA; 2 Department of Mechanical Engineering, University of Washington, Seattle, WA 98195, USA; 3 Harborview Medical Center, Seattle, WA 98104, USA

**Keywords:** surface electromyography, stroke, neurorehabilitation, upper extremity, case study, acute care, sub-acute rehabilitation

## Abstract

Arm recovery varies greatly among stroke survivors. Wearable surface electromyography (sEMG) sensors have been used to track recovery in research; however, sEMG is rarely used within acute and subacute clinical settings. The purpose of this case study was to describe the use of wireless sEMG sensors to examine changes in muscle activity during acute and subacute phases of stroke recovery, and understand the participant’s perceptions of sEMG monitoring. Beginning three days post-stroke, one stroke survivor wore five wireless sEMG sensors on his involved arm for three to four hours, every one to three days. Muscle activity was tracked during routine care in the acute setting through discharge from inpatient rehabilitation. Three- and eight-month follow-up sessions were completed in the community. Activity logs were completed each session, and a semi-structured interview occurred at the final session. The longitudinal monitoring of muscle and movement recovery in the clinic and community was feasible using sEMG sensors. The participant and medical team felt monitoring was unobtrusive, interesting, and motivating for recovery, but desired greater in-session feedback to inform rehabilitation. While barriers in equipment and signal quality still exist, capitalizing on wearable sensing technology in the clinic holds promise for enabling personalized stroke recovery.

## 1. Introduction

Stroke is among the most frequent and costly causes of new onset disability in adults [1-3]. About half of all stroke survivors report challenges in upper extremity motor function six months post-stroke, which can include hemiparesis, spasticity, co-contraction, pain, or other limitations which impact quality of life [3-6]. While ongoing research continues to explore areas of brain plasticity, neural recovery mechanisms, and prognostication of stroke outcomes to maximize recovery, there has been simultaneous advancement in wearable sensor-based technologies that provide additional, noninvasive means of examining neuromuscular pathways and recovery processes following stroke [7-10]. Several wearable sensor options exist for monitoring recovery in stroke survivors, such as sensing textiles, vital sign or muscle activity electrodes, or inertial measurement units (IMUs), which provide clinicians, researchers, and survivors themselves with valuable information about activity counts, kinematics of the upper or lower extremities during functional tasks or gait, balance responses, and even sleep quality [11-13]. IMUs have been shown to improve the specificity of monitoring and increase objective understanding of subtle recovery metrics or responsiveness to intervention in both lab and clinic settings; however, challenges remain in implementation due to issues such as battery life, nonstandard or proprietary algorithms across sensor brands, comfort, and signal variation in stroke survivor movements following injury [14,15].

Another key wearable technology is surface electromyography (sEMG). Surface EMG sensors detect and record electrical impulses from muscle tissue that reflect the activation of the corticospinal tract, reflexes, and other neural pathways [9]. Scientists have used sEMG to describe directional patterns of upper extremity muscle recovery, examine co-contraction responses and spasticity, describe changes in latency in muscle response across time, explore firing patterns and interlimb coupling in stroke survivors, and examine the role of sEMG biofeedback to enhance functional rehabilitation outcomes [16-19]. With the ability to be worn for long periods of time, sEMG can continuously monitor activity and identify changes in motor function not otherwise noted during clinical exams [20-22]. Current clinical assessments are observational, making it challenging to detect subtle changes and provide valuable feedback for the patient [23,24]. The diagnostic abilities of sEMG may provide an opportunity to overcome such challenges and obtain a detailed assessment of motor control after stroke and throughout recovery, in addition to a better understanding of the effectiveness of therapies [25]. However, sEMG has not been widely adopted in the clinic, despite published standards for use and research indicating that sEMG could be a useful tool in determining long-term prognosis and rehabilitation planning [9,19,26]. Further, few studies have evaluated rehabilitation technologies, such as sEMG, during acute and subacute phases of stroke recovery, with the bulk of the literature engaging chronic stroke survivors [27]. Qualitative exploration of perceptions of wearable sensors in clinical settings has also been limited, aside from the previous work of this research group [28,29]. In the midst of conducting two larger studies, one that incorporated qualitative methods with stroke survivors in the community, and one that focused on quantitative sEMG tracking with a different group of hospitalized participants, the research team had the unique opportunity to perform both sets of research procedures with one participant who was able to be followed through acute hospitalization, subacute recovery in inpatient rehabilitation, and following discharge, due to remaining in the same medical system throughout recovery. Thus, the purpose of this case study was twofold: (1) describe and provide proof-of-concept for the use of wireless sEMG sensors to track changes in muscle activity during acute and subacute phases of stroke recovery in a single patient, and (2) understand the perceptions and perceived benefits and drawbacks of real-time muscle activity monitoring from the participant’s point of view across the span of recovery.

## 2. Materials and Methods

### 2.1. Participant

The participant, Jack (a pseudonym), was a previously healthy 56-year-old Caucasian, right-handed male with a right caudate body and lentiform nucleus ischemic stroke, likely of small vessel disease etiology. He was not a candidate for tissue plasminogen activator or thrombectomy. His exam was notable for left hemiparesis, left facial droop, and dysarthria. His total NIH Stroke Scale score was 10, with scores of 4 for both the left upper and lower extremities. Further clinical assessment revealed intact sensation to light touch in all extremities, right gaze preference, and a Modified Ashworth Scale score of 1 for left elbow flexion and extension and left wrist extension, suggesting a slight increase in muscle tone. However, no spasms or hypertonicity were documented thereafter. Rehabilitation services were initiated on hospital day zero and continued throughout his acute care stay and subsequent transfer to inpatient rehabilitation. Motor function was evaluated with manual muscle testing (MMT) throughout the 4-day acute hospital course, in which Jack exhibited motor recovery of his left leg, but little improvement of his left arm. On average, Jack participated in occupational and/or physical therapies two to four times per week during his acute course.

**Figure 1.**
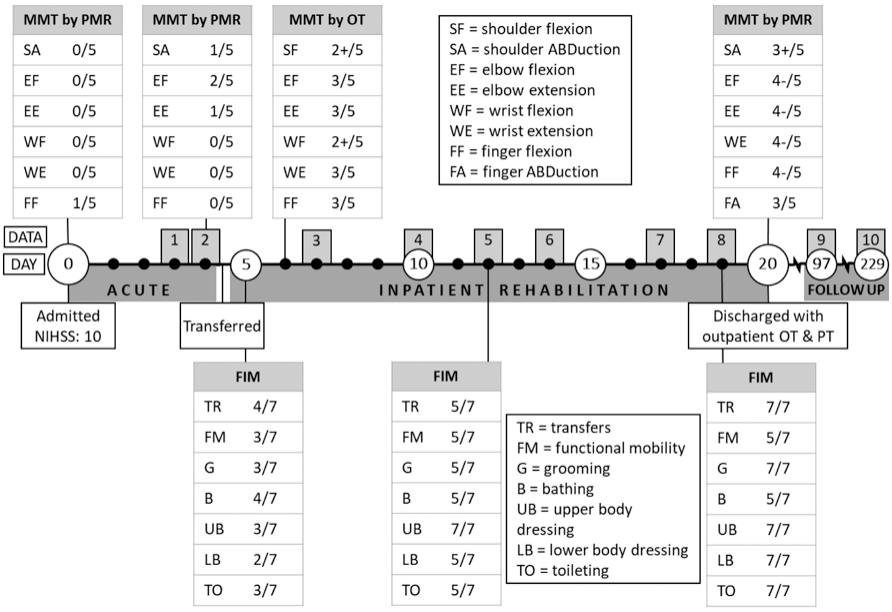
Timeline of acute and inpatient rehabilitation with functional clinical assessment scores. This figure denotes the timeline of the participant’s recovery and data collection across 10 visits. Manual muscle testing (MMT) and functional independence measure (FIM) scores were obtained from the medical record as documented by the physical medicine and rehabilitation (PMR) physician or occupational therapist (OT) on days noted. Major events for data collection, functional scoring on outcome measures, and clinical care are shown above and below timeline.

Subsequently, Jack was transferred to the inpatient rehabilitation unit of an affiliated hospital with left hemiparesis, impaired coordination, impaired balance, and dysarthria. He was fitted with a GiveMohr® sling (Albuquerque, NM, USA) for mild shoulder subluxation and pain. On average, Jack spent about 90 min in occupational therapy (OT) and 150 min in physical therapy (PT) per day, five to seven days per week. Over the 16-day course of inpatient rehabilitation, he had significant motor recovery in his left upper extremity and improvement in fine motor coordination and initiation of motor movements, as documented by traditional clinical measurements and medical records (Figure 1 and Table 1). At his three-month follow-up session, Jack was back to working part-time in the corporate financial sector, attending outpatient OT and PT one to two times a week, and using a verbal dictation typing program as a workplace accommodation. He was exercising about three times per week and performing endurance and strength training on his own. At his eight-month follow up, Jack had returned to two-handed typing, was working full time, had been discharged from OT and PT services, and was exercising at a similar frequency to his previous follow up.

As Jack remained within the same hospital system for acute and inpatient recovery, he was the only participant who took place in this research group’s hospital-based sEMG study as well as a community-based qualitative study with stroke survivors following his discharge, and was the only participant in either study to have experience with sEMG monitoring in both settings. Thus, the study team capitalized on the ability to synthesize both sEMG outcomes and qualitative responses for this single participant.

### 2.2. Intervention

**Figure 2.**
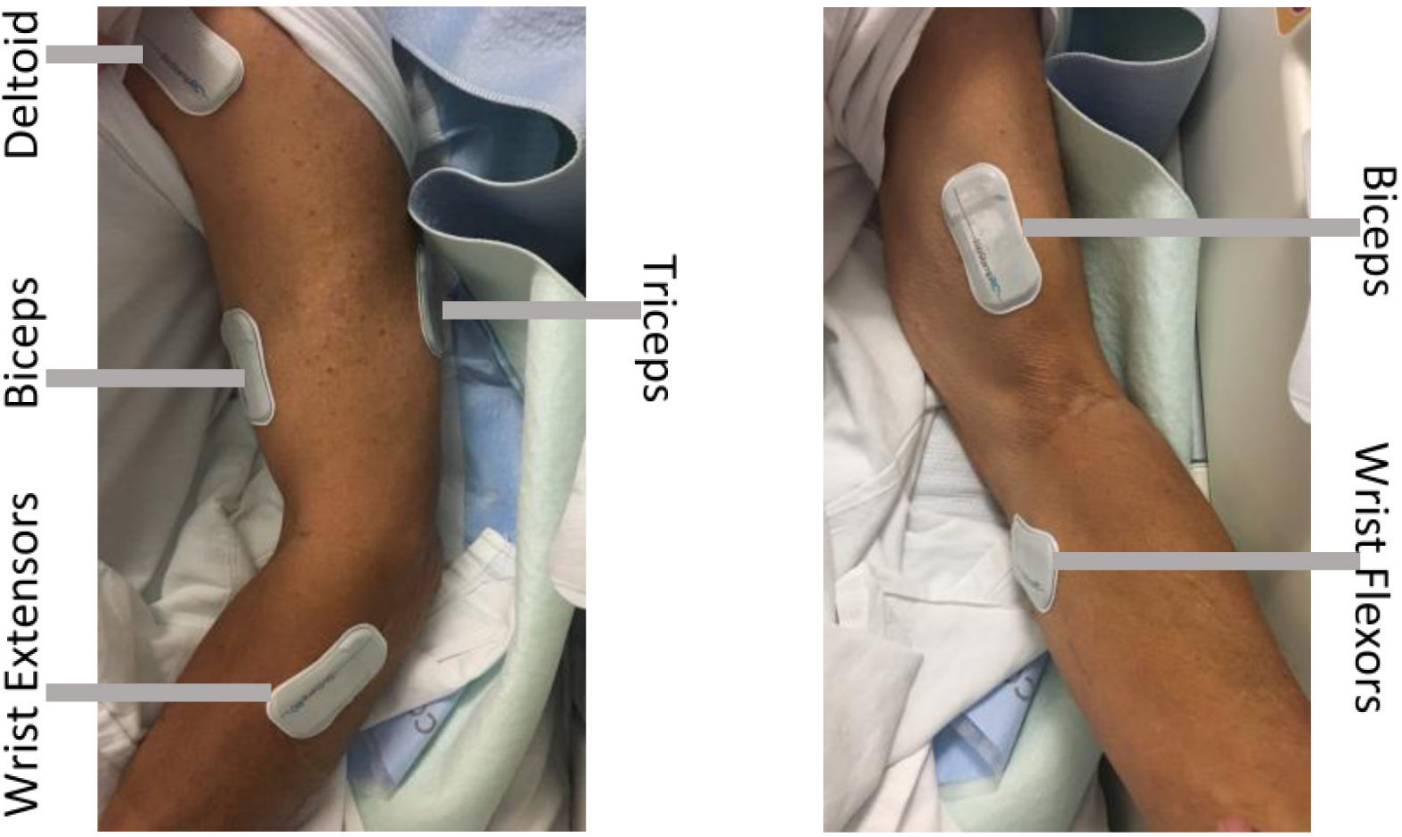
Sensor placement.

All study procedures were conducted with approval by the authors’ institutional review board and with informed consent from the participant. A standard protocol was used for all study procedures to ensure repeatability across this study as well as in future work. A set of five wireless, water-resistant sEMG sensors (Biostamp RC®, MC10 Inc., Lexington, MA, USA) were placed on the participant’s left upper extremity. Each sensor was 6.6 × 3.4 × 0.3 cm^3^ in a housing made of a flexible, low-durometer silicone. These sensors were selected from many research lab-owned systems due to their low profile, flexibility, remote monitoring capabilities, and ability to sanitize in-hospital environments [28,29]. Prior to placement, the skin was prepared by washing with soap and water, and electroconductive gel was applied to each sensor. Sensors were placed at the anterior deltoid, long head of the biceps, and lateral head of the triceps according to the Surface Electromyography for the Noninvasive Assessment of Muscles (SENIAM) guidelines standard placement procedures [30]. Bony anatomical landmarks of the medial and lateral humeral epicondyles were used for standard placement of the subsequent two sensors at the wrist flexor and extensor muscle groups, as SENIAM does not offer placement guidelines for these muscles (Figure 2). To further ensure consistency, the same researcher placed the sensors at each data collection visit. Due to the size of the sensors, only large muscles and groups could be observed. The sensor placement allowed for the observation of proximal and distal muscle activation. The setup and placement of the sensors took approximately 10 min. Individual serial numbers were used to consistently pair the same set of sensors and muscles.

Muscle signal data were collected for three to four hour sessions every one to three days, beginning on post-stroke day three through discharge from inpatient rehabilitation. Session length was limited by the local memory storage allowance (32 MB) of each sensor. During each session, a researcher documented activity at 30-min intervals, but did not remain in the room throughout data collection, so as not to interfere with regularly scheduled activities or rest (Table 2). Two additional follow-up sessions in the community were conducted at three and eight months post-stroke, at the participant’s place of work. The same protocol was used for sensor placement and data collection in the community for consistency, with the exception of Jack tracking his activity (e.g., desk work or exercise) himself and reporting this to the researcher. Each of the community follow-up sessions included a period of exercise as well as sedentary activity.

Figure 2 Sensor placement of BioStamp electrodes on the participant’s left deltoid, biceps, triceps, wrist extensors, and wrist flexors. Beginning at sEMG session 3, the forearm and upper arm sensors were wrapped with Coban to prevent sensors from falling off during therapeutic activities.

EMG data were recorded at 1000 Hz with a resolution of 0.0006 mV, range of ±200 mV, and no hardware signal processing. Data processing and analysis were performed in MATLAB® (MathWorks, Inc., Natick, MA, USA). Missing or faulty data were removed by comparing signal data to the data collection log (e.g., a sensor fell off or did not record). Data were bandpass filtered (20–400 Hz), conditioned with a Teager–Kaiser energy operator, rectified, and low-pass filtered (50 Hz, 4th order Butterworth) [31,32]. Signal baseline was identified over the collection period using a gliding window to locate sections with the lowest variance (window length: 10 s; step: 0.5 s). The threshold was then computed as a summation of the baseline average and average of the baseline standard deviation multiplied by a preset value of 6 for the threshold level, similar to prior sEMG research using the Teager–Kasier energy operator [33]. Data were then categorized into three types of activity: “In Room”, “In OT”, or “In PT”, and further classified based on contraction duration. Analysis focused on contractions 100–500 ms in length to exclude false contractions due to sensor noise or movement artifact. Within this contraction length range, three primary comparisons were made between activities: average contractions per minute, median amplitude, and median contraction length (MS). Average contractions per minute provided insight into how active the muscle was during recorded activities, while amplitude represented the force generated by the muscle. Contraction length provided insight into endurance, fatigue, and potential presence of spasticity. All of these metrics represent valuable and potentially actionable clinical information during acute stroke recovery.

During the 8-month follow up in the community, a semi-structured, in-depth interview was also conducted to understand Jack’s perceptions of recovery, rehabilitation, and benefits and drawbacks of his experience with sEMG. The interview was audio recorded, transcribed verbatim, and coded by the research team until 100% agreement was reached for emergent themes. (Table 3).

## 3. Results

Ten data recordings were captured from late January through late September of 2018. Two sessions occurred in the acute hospital setting, six during inpatient rehabilitation, and two in the community at three and eight months post-stroke, consecutively, for a total of 34 h and 17 min of data recordings. The quantitative and qualitative results that follow are presented chronologically, describing (1) acute recovery, (2) inpatient rehabilitation, (3) recovery at home, and (4) perceptions of sEMG use.

### 3.1. Acute Recovery

**Figure 3.**
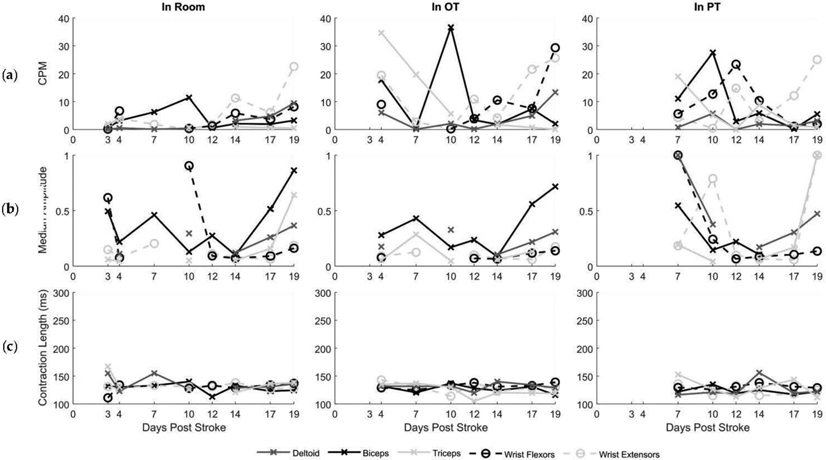
Muscle activity measured by contractions per minute ^a^, mean amplitude ^b^, and contraction length ^c^. Activity of five upper extremity muscles from the participant’s affected limb were tracked every 1–4 days during acute and inpatient rehabilitation. Muscle activity was evaluated based on activity type: ‘In Room’, ‘In OT’, and ‘In PT’ (columns). Contractions between 100 and 500 ms in length were analyzed in contractions per minute (CPM) (**a**), median amplitudes normalized across activity type (**b**), and by contraction length (**c**). Missing data are due to therapy schedules, sensors falling off participant, or sensor failure.

**Figure 4.**
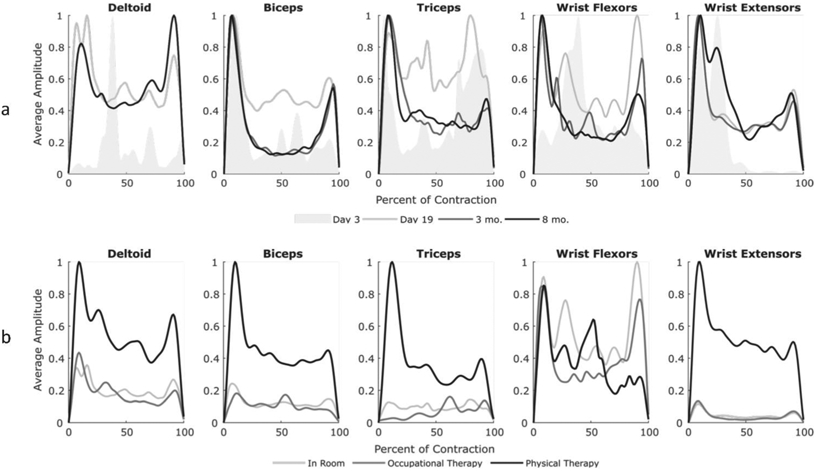
Average amplitude of muscle contractions across days and activities. (**a**) Average contraction profiles normalized by length of contraction and the maximum of each visit. The standard deviation of the contraction profiles for the first recorded session on Day 3 is shown as a shaded region, while Day 19 and the three- and eight-month follow up are the average profiles. The deltoid did not have data for the three-month visit as it fell off the participant. (**b**) Average contraction profiles during Day 19 of inpatient rehabilitation normalized by each visit to compare between activity types. The deltoid, biceps, triceps, and wrist extensors exhibited similar average contraction shapes as well as relative amplitudes between activity types.

Jack’s description of his mobility during the early days following stroke correlate with his MMT scores (Figure 1 and Table 4, Quote 1). Similarly, the examination of muscle activity showed a low number of contractions, consistently at or below ten contractions per minute for all muscle groups when not in therapy (Figure 3a). Examining average contraction profiles, at Day 3, muscles typically demonstrated only a single burst of activity at lower amplitudes, especially for the deltoid, biceps, and wrist extensor groups (Figure 4a) during acute recovery. The low amplitude generated by the few contractions corresponded to Jack’s initial inability to move his arm. Nonetheless, muscle activity was detected with sEMG, despite no observational movement by either Jack or the clinicians.

### 3.2. Inpatient Rehabilitation

Although Jack continued to experience significant functional limitations at the end of inpatient rehabilitation, improvements were noted in strength and his ability to complete tasks (Figure 1 and Table 4, Quote 2). Additional qualitative functional improvements were extracted from the medical records, but quantitative assessments were limited (Table 1). These clinical findings correlate with an increase in the median amplitude of muscle activity in all tested muscle groups, with the exception of the wrist extensors during in-room and in-therapy activities (Figure 3b). Examining average contraction profiles (Figure 4a), the participant demonstrated more sustained contractions at the end of inpatient rehabilitation (Day 19), especially for the deltoid, biceps, and wrist extensors compared to Day 3. Instead of the initial burst seen during acute recovery, muscles generated greater force throughout the contraction. Interestingly, average contraction lengths did not vary between acute recovery and inpatient rehabilitation, with a median duration of 130 ms (Figure 3c). The deltoid, biceps, triceps, and wrist extensors exhibited similar average contraction shapes and relative amplitudes between in-room and OT activities at discharge, while PT typically exhibited twice the scaled amplitude (Figure 4b). The increased time in PT and focus on gross motor control activities could account for this difference.

### 3.3. Recovery at Home

**Figure 5.**
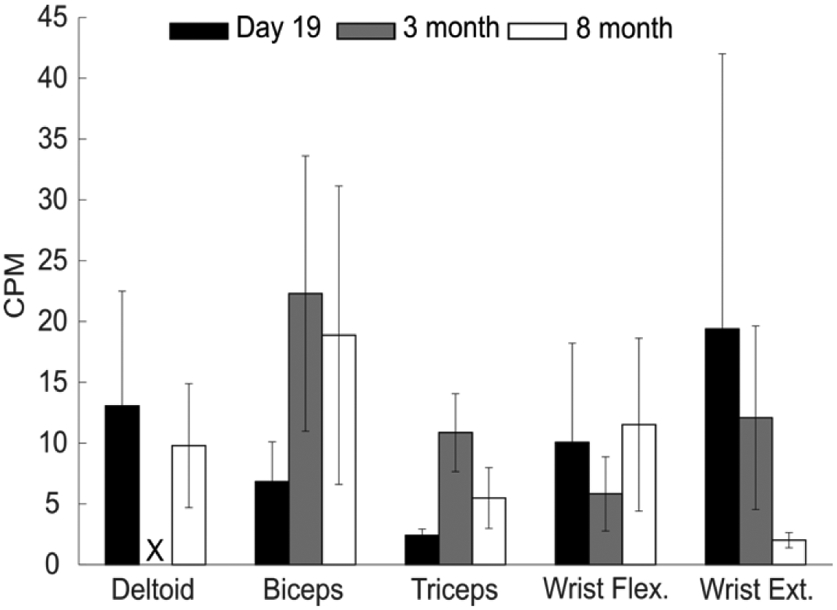
Contractions per minute by muscle group at discharge and follow up. Average contractions per minute during the last in-patient rehabilitation day and the three- and eight-month follow-up visit. Activity type for each session involved no therapy or workout but involved the participant’s daily activities. The vertical lines represent standard deviation, and no contractions between 100 and 500 ms were recorded for the Deltoid at the three-month follow up (X).

Jack continued with outpatient physical and occupational therapy after discharge, noting continued progress in his everyday functional abilities (Table 4, Quote 3). After eight months, he reported continued improvements, noting strength gains of 60 lb of left-hand grip force, as measured with a grip dynamometer, and quantifying his overall recovery at 70% (Table 4, Quote 4). However, the examination of his average contraction profiles revealed similar patterns of sustained contraction at the long-term follow-up sessions as those recorded at discharge from inpatient rehabilitation (Figure 4a). Average contractions per minute across all muscle groups between discharge and community follow-up sessions varied but trended upward in the biceps, triceps, and wrist flexors (Figure 5).

### 3.4. Perceptions of sEMG Use

Both the participant and the medical staff engaged in his care were interested in detecting muscle signals in the involved upper extremity over time and appreciated real-time muscle signal and acceleration graphs on the Biostamp RC® tablet. The participant would have preferred if the tablet remained in proximity, alerted him when a new threshold was reached, or better described what the graphs represented in terms of his recovering muscle activity, especially in noting subtle changes between sessions (Table 4, Quote 5). Jack voiced that he would have preferred a cell phone to access the sEMG sensor information, because “Everyone always has it”. While Jack expressed a desire to use the sEMG sensors after he transitioned home to document progress during daily tasks, he noted he would have needed help putting them on (Table 4, Quote 6). Despite these challenges, Jack indicated that further clinical information from sEMG technology would be motivating, even if the changes observed were subtle (Table 4, Quote 7).

## 4. Discussion

Though the scope is limited, this case study is among the first to describe both the proof-of-concept of the preliminary implementation of commercial sEMG sensors directly in clinical and community settings to monitor upper extremity muscle activity in the acute and subacute phases of rehabilitation, and report the participant’s experiences of recovery and perceptions of such sEMG monitoring after stroke. This study presented a unique opportunity to describe sEMG monitoring results together with qualitative participant perceptions of recovery and monitoring in a single individual who took place in two sets of study procedures. Although previous work has examined the return of finger extension mobility beginning at 14 days post-stroke, or in subacute populations, most frequently studies incorporating sEMG examine upper extremity recovery of nonhospitalized chronic stroke survivors [17,34-36]. The findings of this study indicate that the application of sEMG in acute recovery is feasible, and reflects recommendations in the previous literature indicating sEMG and other stimulation/biofeedback technology as both a means to detect and train the early return of limb movements and as a potential prognostic aid in directing stroke rehabilitation [9,37,38]. This is especially relevant given that current clinical rehabilitation assessments lack the ability to differentiate between true muscle recovery and compensation [24,38,39]. Changes in muscle activity detected by sEMG combined with observable improvement in function offer a more objective means to make this differentiation and potentially enhance stroke recovery outcomes. Further, since the return of finger and hand movements has been highlighted as a critical indicator of functional prognosis in stroke survivors, monitoring with sEMG can be an especially relevant tool for rehabilitation professionals, even prior to the visible active return of movement [35].

Jack’s clinical assessments showed linear improvement, but the metrics from sEMG were highly variable. This may indicate that compensation was responsible for some of the improved functional scores during hospitalization [24]. While in the room, he showed an increase in contractions per minute over time, which points to the benefits described in the literature of sEMG monitoring outside of scheduled therapies or assessments, particularly to help mitigate learned nonuse behavior [22,25]. While there is a lack of published data on contractions per minute expected in healthy or impaired adult upper extremities during functional tasks, one study used rates between 60 and 120 to evaluate sEMG signal properties at the wrist in healthy individuals, suggesting that Jack’s maximal recorded rate of approximately 40 likely indicated residual functional limitation as recovery progressed, which was corroborated by his qualitative interview findings [40]. Nonetheless, this information can be useful to both the clinician and the patient, with prior research indicating that more objective feedback serves to motivate participation and improve self-efficacy during rehabilitation [23]. In this study, Jack similarly expressed a desire for greater real-time feedback from the sEMG and easier access to long term data trends. Finally, this feasibility study points to the value of combining quantitative metrics with qualitative inquiry to better understand how patient motivation and technology acceptance contribute to stroke recovery. Technology acceptance has only been minimally explored in the literature to date, though much rich data describing stroke survivor experiences are available [8,41,42].

It was feasible to set up and run three- to four-hour recording sessions during the participant’s routine clinical care in both acute and inpatient environments with minimal to no interference in routine care. A structured inpatient rehabilitation schedule made it easier to apply the sensors and capture therapeutic activities, compared to acute care scheduling that often varied based on therapist availability and medical procedures. The variability in schedule and different therapeutic activities may have impacted sEMG outcomes. However, it is advantageous to use sEMG to record and track this variability as it may more accurately reflect typical trajectories of arm use and motor control during recovery [43]. What is less clear is whether this type of sEMG monitoring can be reliably conducted in home environments by stroke survivors and their caregivers. However, a researcher or clinician scientist could incorporate training sessions at home to ensure that sensors were reliably placed, in addition to using methods such as temporary tattoos or a fabric sleeve with pre-measured sensor cutouts for individuals to achieve consistency. This type of training would be essential to maximize the clinical utility of in-home sEMG monitoring.

There were several limitations to this study. First, there was a lack of consistent quantitative data in Jack’s electronic medical record that described functional recovery. The available data were reported and compared with sEMG output. Despite purposefully selecting the Biostamp sensors for their technical features among many other systems previously researched by this study team, there were also limitations in the sEMG sensors themselves, pointing to important needs for future development and research [28,29]. While the design of the sensors was comfortable and unobtrusive to the participant, the size of the sensors may have allowed for cross talk between muscles and decreased the quality of the signal. Recordings were affected by occasional failure of the double-sided stickers provided by the manufacturer, especially when the participant was sweating. Similarly, the participant’s changing skin conditions, hydration, and ambient environmental conditions likely influenced skin impedance and data quality, limiting comparisons of absolute sEMG magnitude between days. The hardware also had several limitations. At high sampling rates (1000 Hz), the Biostamp sensors have a noise signal with a frequency of approximately 8.2 Hz which made the analysis of contractions of less than 100 MS impossible. Similarly, while longer periods of monitoring would have been ideal, the data storage capacity and upload time limited the length and frequency of collection. Changes in the participant’s upper extremity muscle tone throughout the duration of the study may have impacted the consistency of sensor placement by the researcher, although SENIAM placement guidelines were followed by the same researcher at each visit to maximize consistency [44]. While Jack was quite motivated throughout his recovery and demonstrated significant functional return, his recovery process may not be representative of a typical stroke survivor, and must be considered only as a distinct experience of individual recovery. However, this does not diminish the applicability of the study procedures and proof-of-concept for sEMG use in hospital settings for other stroke survivors. Finally, the role of the first author as the participant’s primary contact during muscle activity tracking and his interviewer may have led to acquiescence bias or researcher bias during the qualitative interview. However, this in-depth engagement also allowed the establishment of a rapport and trust between the researcher and participant over time, serving as a potential mitigating factor [45].

Future research must broaden the context of this case study to track sEMG data and qualitative responses on recovery and muscle monitoring over time in a larger and more diverse sample of participants. Additionally, repeated-measure monitoring at multiple points in the same day could be useful to allow the direct comparison of muscle activity and sensor reliability. As stroke recovery is a highly complex process, equally complex analyses should also be implemented in future work that explore the role of sEMG in monitoring recovery. For example, coherence analysis, which has been used to investigate neural mechanisms of muscle activity through linear correlation, has been examined in sEMG-intramuscular EMG and sEMG and electroencephalogram pairings, or in sEMG rectification processes [46,47]. This same analysis could be applied to muscle firing rates, co-contraction, or coordination pattern pairings during functional movement in recovery. Similarly, frequency domain analysis is used to make inferences on motor unit performance, assessing parameters such as the conduction velocity of muscle fibers and muscle fatigue, which are also useful to understand the trajectory of clinical recovery [48,49]. While these metrics were outside the scope of this case study exploring the proof-of-concept of sEMG monitoring combined with the participant perception of muscle activity monitoring in acute and subacute recovery, it is important to consider these analyses to more deeply understand the progression of recovery in future work with new or current datasets. Finally, as new sEMG technologies emerge and existing systems are refined, it is important to assess these within real-time clinical environments, to further understand their acceptability to patients and medical care teams and their utility in clinical decision making during stroke recovery.

## 5. Conclusions

This study demonstrates that using sEMG sensors to clinically monitor upper extremity muscle activity during acute and subacute stroke recovery is feasible, and preliminary outcomes suggest that changes in muscle activity are observed that track quantitative and qualitative changes in function. Wireless sEMG sensors have the potential to significantly improve tracking and training activities in neurorehabilitation following stroke and may be able to aid in long-term prognostication or improve targeted interventions to promote functional outcomes. Surface EMG technology may also offer hope and motivation to stroke survivors during their early recovery, provided they have access to a simple, intuitive user interface as well as appropriate feedback and education on the signals measured and how they correlate to function. However, current limitations in technology, processing time and procedures, and the user interface must be addressed to provide more useful and valuable information to both clinicians and users. Future studies should examine qualitative and quantitative outcomes in greater numbers of participants with diverse post-stroke presentations, and the further trial of other types of wearable sEMG sensor technologies should be undertaken in healthcare and community settings. Further research should also consider which sEMG metrics most accurately reflect recovery.

## Figures and Tables

**Table 1.** Functional assessment documented in EMR.

Days Post Stroke	Qualitative Functional Assessment
7	Patient demonstrated progress but also having difficulty with engaging left upper extremity fingers … Patient presented with emerging 2 point pinch and full palm grasp and release today.
10	Patient showing increased function in his left upper extremity. Fine/gross motor activity with great improvement and effort compared to previous session.
12	Patient participated in fine and gross motor strengthening and coordination with great effort. Demonstrating great key pinch and emerging pincer pinch.
14	Continues to demonstrate good progress with left upper extremity function performing exercises with great effort and improved control. Continues to need more work on wrist extension, thumb abduction, and middle/ring finger control. Difficulty with coordinating movements.
17	Patient presented with improved left upper extremity function. Increased coordination compared to previous session.
19	Patient demonstrated great progress again. Participated in fine motor activity with great effort and minimum cues for coordination.

**Table 2.** Activity log during sEMG recordings.

Data	Total Recording Time	In Room		In OT		In PT	
		Time	Activity	Time	Activity	Time	Activity
Day 3	192 min	192 min	Resting in bed				
Day 4	184 min	161 min	Sitting in wheelchair	23 min	Range of motion and strength testing		
Day 7	186 min	66 min	Back in room	60 min	Fine motor coordination in pinch and grasp, passive and assisted range of motion, mirror box, and e-stim	60 min	Therapeutic functional activity/bed mobility
Day 10	250 min	135 min	Eating lunch and napping	60 min	Therasponge and theraputty for L hand	55 min	Therapeutic functional activity/bed mobility, gait/stair training
Day 12	240 min	120 min	Eating lunch and napping	60 min	Self-care/ADL management- cued to use L upper extremity as much as possible	60 min	Therapeutic exercise/procedure, gait/stair training
Day 14	232 min	112 min	Eating lunch and napping	60 min	Left upper extremity exercises of thumb, finger, and wrist	60 min	Shoulder flexion with towel, balance/vestibular training
Day 17	222 min	102 min	Eating lunch and resting	60 min	Self-care/ADL management, left upper extremity finger exercises	60 min	Core strengthening and balance, upper extremity mirror therapy
Day 19	206 min	86 min	Small group conference	30 min	Self-care/ADL management, fine motor activity to increase coordination and endurance	90 min	Balance/vestibular training

**Table 3.** Sample semi-structured interview questions.

	Tell Me about What You Remember about Having your Stroke.
**Sample Interview Questions**	What were your initial goals for recovery? Did your goals for recovery change over time? Describe what it was like for you to wear the sEMG sensors in the hospital. What kinds of information do you wish you could have received while wearing the sensors? In what way would you have preferred receiving such information? (i.e., visual or auditory signals, via cell phone or tablet, written report, etc.) Why might it be beneficial/detrimental to track muscle signals with sEMG during recovery from stroke? In which settings might using sEMG to track muscle activity be most useful? (i.e., home/community vs. hospital or rehab)

**Table 4.** Representative participant responses.

Quote	Theme/Topic	Participant Quote
1	Acute Recovery	“So when I had my stroke, um, it was pretty shocking. I knew what it was, I’ve had enough first aid training to know the signs. I knew I was having one, though I was still surprised … I was in the (first) hospital for five days, and had absolutely no motion in my arm, my hand, or my shoulder, on the left side.”
2	Inpatient Rehab	“The thing about rehab is you, you start to learn that it can become pretty routine. And they can actually set you up for doing a lot of stuff on your own. So you have to be very motivated to do that. Motivation was not a problem for me. Early on, because (I) had the time, and I had the drive to want to use, particularly, my arm and hand much more than I could … I told (the second) hospital that I wanted two plus weeks of rehab. By the time I left, I could walk on my own with a cane … I didn’t have, I had very, very limited motion in my arm, and my shoulder, and my hand. But their goal was to make me self-sufficient.”
3	Recovery at Home	“The arm took a long time, a frustratingly long time. When I went back to work, I still had to get help via software to type. I couldn’t use my hand … I could lift my arm and shrug my shoulders but the fingers itself wouldn’t work. And I was given a lot of home exercises, I would start my days doing all that. At least an hour or two of home exercises, um, pretty religiously too. And for the most part I tried to do normal things. I tried to do dishes, fold clothes, mow my lawn, clean the house … we played a lot of board games, and I would totally use my left hand for everything, which wasn’t normal for me, but was good for that … recovery. And it got better and better, you know, to the point where for the first time I could cut a piece of meat with a fork and a knife. It was pretty exhilarating, that was a big celebration, even though (laughing) my hand would still dip, like, into my horseradish sauce eating prime rib!”
4	Recovery is Ongoing	“(When people ask me how far I’ve come) I usually answer that in three ways. Totally, about 70%. My leg, about 90%, but there’s still differences and weakness in my knee. Arm … probably 60%. There are days it feels like 90%, and there are days, or times in a day, where it feels less. I know I’m not 100%, and I may not be either, and I’m okay with that. You know, cause I can walk. I can run, I can talk. But the things that I notice now, they’re subtle… subtle to most people, but they’re very noticeable to me.”
5	Perceptions of sEMG Use	“I was intrigued … however, I don’t know what all the readings tell you, I mean, so that is of interest, what you all were seeing … I didn’t dislike anything though, but what I thought was compelling about it was everybody told me that this (hand function return) would be slow. Well, guess what? Eight months later, this is still recovering. So I was, I was hopeful that it would show signs of things that are occurring when I couldn’t physically feel it … if you had other scientific evidence that things were happening, even beyond their notion that it would, it gives you a lot of hope. You just have to be patient, and it’s harder to take when someone tells you, but easier to understand if someone actually shows you.”
6	Limitations of sEMG Sensors	“When you and I got together, it was a lot to take on and off. That’s kind of a pain, right? I’m wondering if there is a way to do kind of both. That … that has multiple individual muscle sensors where you pull a sleeve on, for example. As long as you align it correctly, it’s getting a, a number of muscles.”
7	sEMG as a Motivator for Improvement	“For the most part when I was at my, my worst, I couldn’t tell if things were really going differently, but maybe it was cause it was so subtle. Cause I want big changes or I want big improvements. But again seeing some improvement, whatever scale, scientifically with your data, could be a big boost. Because there were times where I can tell no difference at all, but I’m sure there was something there. And at home, you’re doing this on your own, that’s the longer-term harder stuff. If you have a way of telling that at home, it’s kind of nice to get that affirmation through any means you can.”

## Data Availability

Data from this study available upon request.
